# Supplementary material for: Burden and factors associated with ongoing transmission of soil-transmitted helminths infections among the adult population: A community-based cross-sectional survey in Muleba district, Tanzania
Source: PLoS One. 2023 Jul 26;18(7):e0288936. doi: 10.1371/journal.pone.0288936 (PMC10370771; doi:10.1371/journal.pone.0288936)
Supplement: Attachment — Submitted filename: letter to reviewers and editor.docx [file pone.0288936.s003.docx]

Dear Editor, Dear Reviewers,

Thank you for the last comments to our work. Please find attached the revised version of the manuscript.

We have checked references and fixed the issues detected.

English carries at the same time the blessing and the burden of being a shared communication tool among Scientists in the world. Native speakers could write a perfect English version of our work, but the mindset behind certain language choices, words, length, expression, that reflects the cultural background and identity of authors from other parts of the world, would be lost.

Of course, I hope we managed to fix grammar and major flaws.

On behalf of the authors, I thank again and wish a good day.

Dr. Valeria Silvestri
